# Arthroscopic Suprapectoral Retensioning Biceps Tenodesis

**DOI:** 10.1016/j.eats.2024.102922

**Published:** 2024-02-08

**Authors:** Ziyad El Qirem, Mohamad Makahleh, Amer Jadallah, Barakat Elsaqa, Wael Al-Atout

**Affiliations:** a Department of Orthopedic Surgery, The Specialty Hospital, Amman, Jordan; b Department of Orthopedic Surgery, American University of Beirut, Beirut, Lebanon

## Abstract

Tendinopathy of the long head of biceps is a relatively common pathology that we usually see in most of our shoulder arthroscopy procedures. Surgical treatment for long head of biceps tendinopathies ranges from simple biceps tenotomy to biceps tenodesis depending on many factors, two of which are the age and the patient's level of activity. Various techniques in the literature for biceps tenodesis have been described, such as whether to do it open or arthroscopically, suprapectoral or subpectoral, as well as the type of fixation to be used. However, the optimal option is still debatable. In this Technical Note, we describe an arthroscopic technique for distal suprapectoral biceps tenodesis using a knotless corkscrew anchor that has many advantages, such as being an all-arthroscopic with the ability to retension the tendon after implantation. We support our technique with photos and videos with detailed explanations of the technique.

It has been suggested that the long head of the biceps (LHB) tendon is a postoperative pain generator, and its disinsertion has become an increasingly common surgical procedure among shoulder surgeons. Biceps tenotomy and tenodesis have both been used, with comparable outcomes reported. However, biceps tenodesis has the advantage of avoiding the cosmetic Popeye deformity and possibly better function, which makes it the ideal choice for the younger, more active population.^1^^,^^2^

Multiple techniques for biceps tenodesis have been described. Even though no technique has shown a superior outcome,^3^ many have suggested that the intra-articular tenodesis at the upper part of the bicipital groove might result in residual pain, as it fails to address the diseased distal part of the tendon within the groove and the surrounding tenosynovium.^4^ In contrast, the open subpectoral technique comes with more probable complications of greater blood loss, wound infection, stiffness, or transient neuropraxia.^5^^,^^6^

In this Technical Note, we describe an arthroscopic technique for distal suprapectoral biceps tenodesis that allows retensioning of our fixation using a corkscrew knotless suture anchor. This article received the approval of local institutional review board committee.

## Surgical Technique (with Video Illustration)

### Preoperative Evaluation

Patients with biceps tendinitis usually present with anterior shoulder pain, and their symptoms may be similar to rotator cuff or impingement pain. Bicipital groove tenderness, speed, and Yergason tests are usually positive. Diagnosis can be made with the help of ultrasound or magnetic resonance imaging, as they delineate the pathology in a better way and can be used to evaluate any associated pathologies, such as rotator cuff tears.

### Setup

After general anesthesia and an interscalene block, the patient is placed on the operative table in a beach-chair position. Then, after painting and draping the shoulder, we mark our arthroscopic portals in the operative shoulder.

### Portals

**Fig 1:**
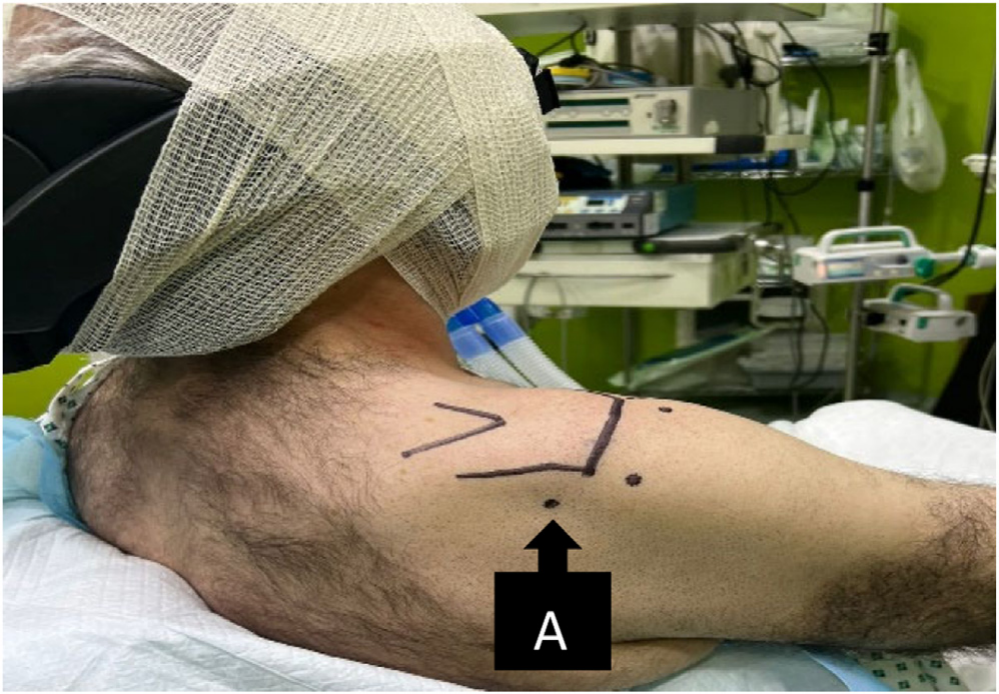
The posterior portal is marked “A.” The patient is in a beach-chair position, and the right shoulder is shown.

**Fig 2:**
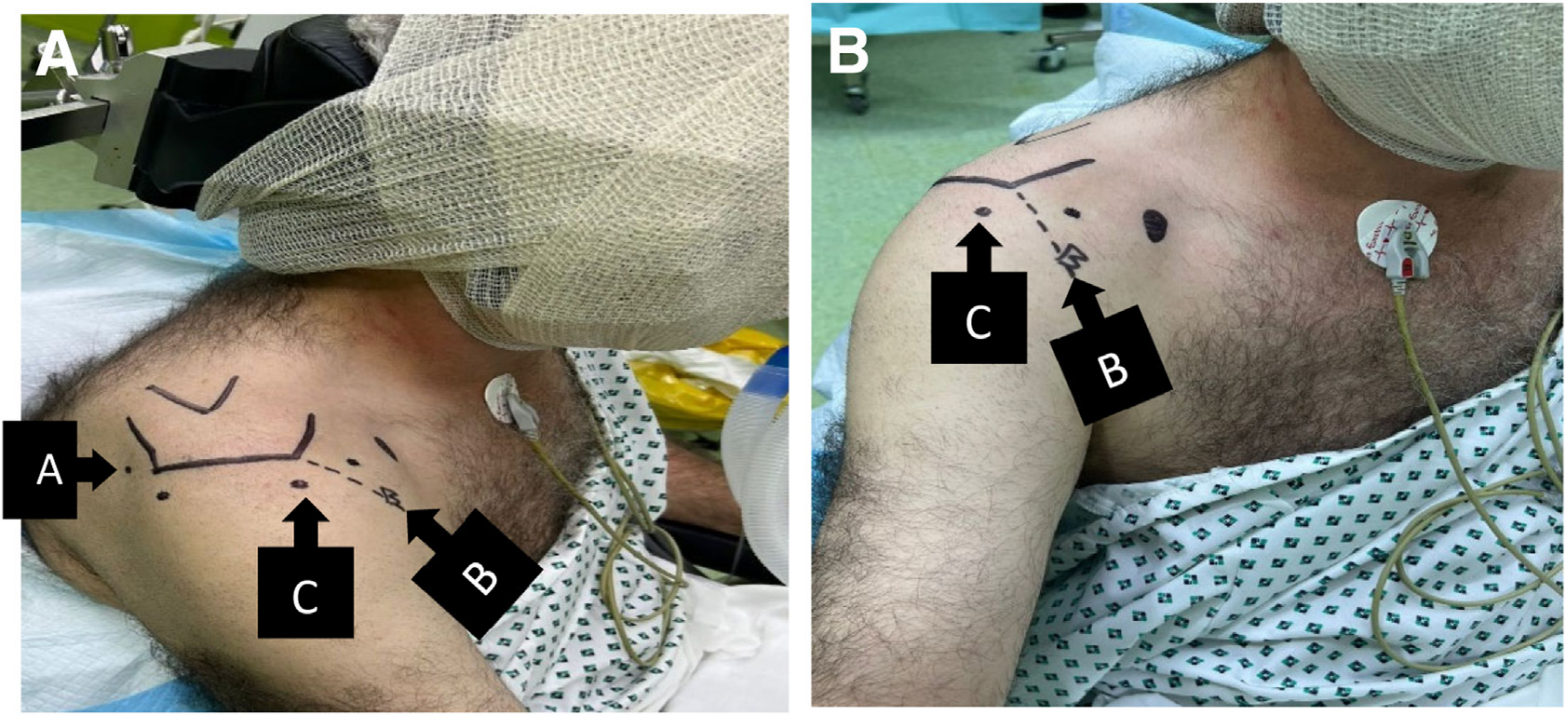
(A and B) The patient is in a beach-chair position, and the right shoulder is shown. “A” Posterior, “B” anteroinferolateral (biceps portal), and “C” anterolateral portals are marked.

This technique is performed using a 30° arthroscope. Four arthroscopy portals to the shoulder are made: standard posterior and anterior portals (diagnostic portals), a high anterolateral portal, and an accessory anteroinferolateral portal (biceps portal) as the main working portal and for anchor placement (Figs 1 and 2 A-B). The operative technique is shown in Video 1.

### Operation

#### Diagnostic Arthroscopy

**Fig 3:**
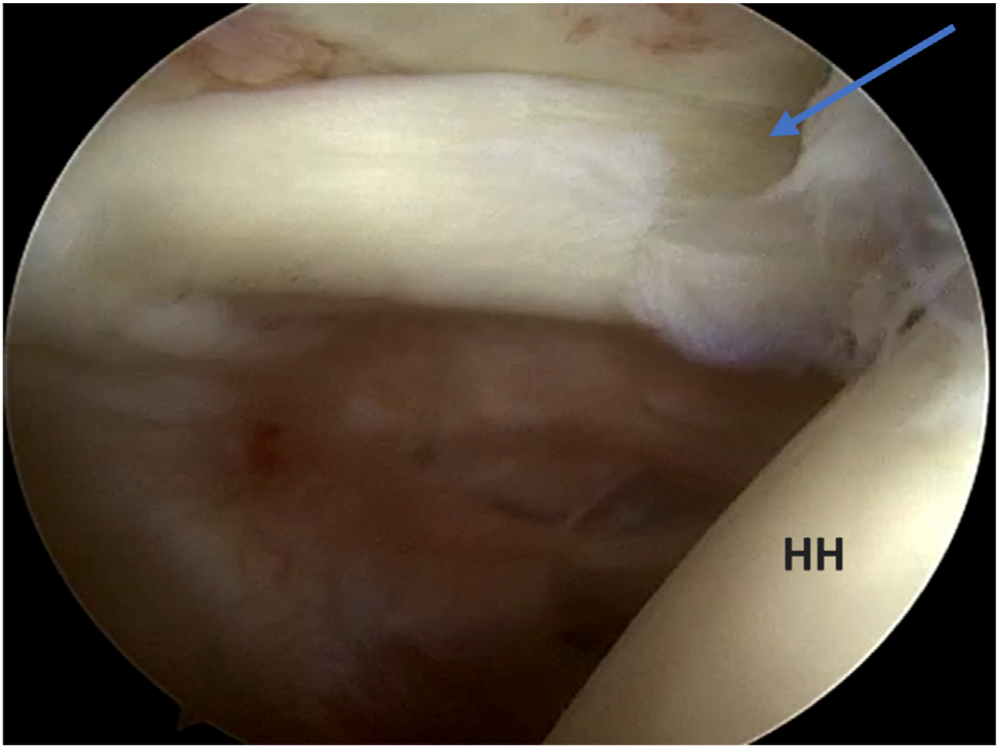
The patient is in a beach-chair position, and the right shoulder is shown. Viewing from the posterior portal, in the glenohumeral joint. Biceps tendinosis is shown (arrow). (HH, humeral head.)

**Fig 4:**
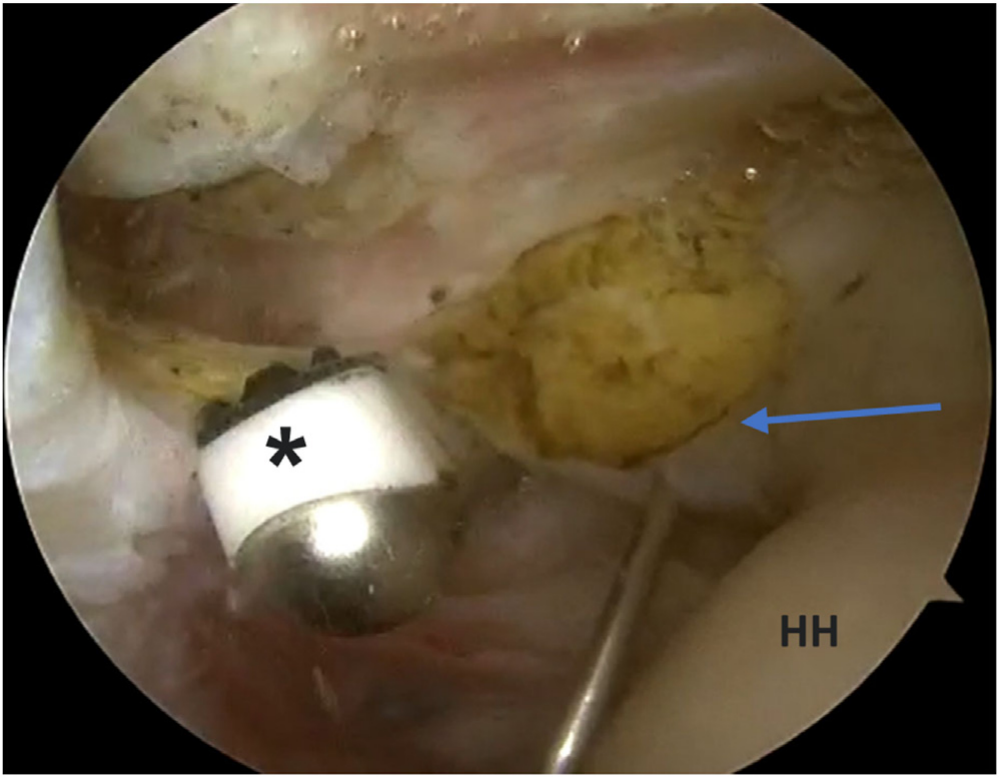
The patient is in a beach-chair position, and the right shoulder is shown. Viewing from the posterior portal, in the glenohumeral joint. An 18-gauge spinal needle is inserted from the anterolateral part of the acromion into the biceps tendon (arrow). Then from the anterior portal, tenotomy of LHB is done using a radiofrequency ablator (Apollo) (asterisk). (HH, humeral head; LHB, long head of biceps.)

A normal diagnostic shoulder arthroscopy is performed. Starting from the standard posterior viewing portal (which is 2 cm inferior and 1 cm medial to posterolateral corner of acromion), we established an anterior portal for probing the biceps tendon, which shows a picture of biceps tendinosis (Fig 3). Once the decision is made for biceps tenodesis, we place an 18-gauge spinal needle percutaneously from the anterolateral part of the acromion to hold the biceps tendon in place (to preserve the length of the biceps). Then, from the anterior portal, we proceed with tenotomy of the biceps tendon near the labrum using an arthroscopic scissor or a radiofrequency ablator (Fig 4).

#### Subacromial Space Preparation

**Fig 5:**
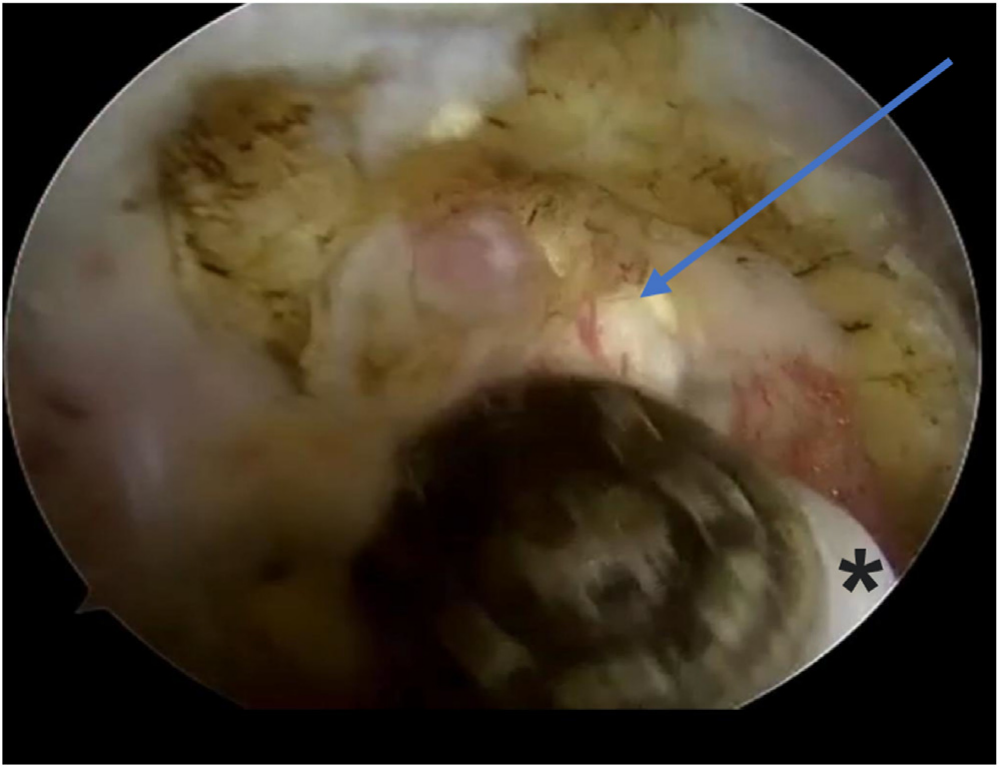
The patient is in a beach-chair position, and the right shoulder is shown. Viewing from the anterolateral portal, in the subacromial space. A radiofrequency ablator (Apollo) (asterisk) is introduced from an accessory anteroinferolateral portal (biceps portal) to release the LHB (arrow) from its groove.

Moving to the subacromial space, shaving of the bursa is done mainly on the anterolateral part. The viewing portal is switched to an anterolateral portal. Flexion at the shoulder joint is done to open up the space anteriorly. An accessory anteroinferolateral portal (biceps portal) is established. From this portal, shaving the anterolateral bursa is done. Then a radiofrequency ablator (Apollo; Arthrex, Naples, FL) is inserted through the biceps portal. It is easier to find the bicipital groove as we go distal to proximal by noticing a thin bicipital groove coverage just above the superior part of the pectoralis major tendon as compared with its proximal aspect. Also, with the help of the Apollo, we can feel a slight depression between the anterior portion of the supraspinatus and the subscapularis, which gives us a hint in locating the groove. To double-check that we are in the correct location, the location of the 18-gauge spinal needle in the subacromial space (that was inserted from the anterolateral part of the acromion) should almost be in line with our bicipital groove location. After that, we open the groove with a radiofrequency ablator (Apollo) at the distal part of the transverse humeral ligament, and we avoid going too much proximal, as we don't want to harm the common tendon between the supraspinatus and the subscapularis tendon (Fig 5).

#### Biceps Tendon Preparation

**Fig 6:**
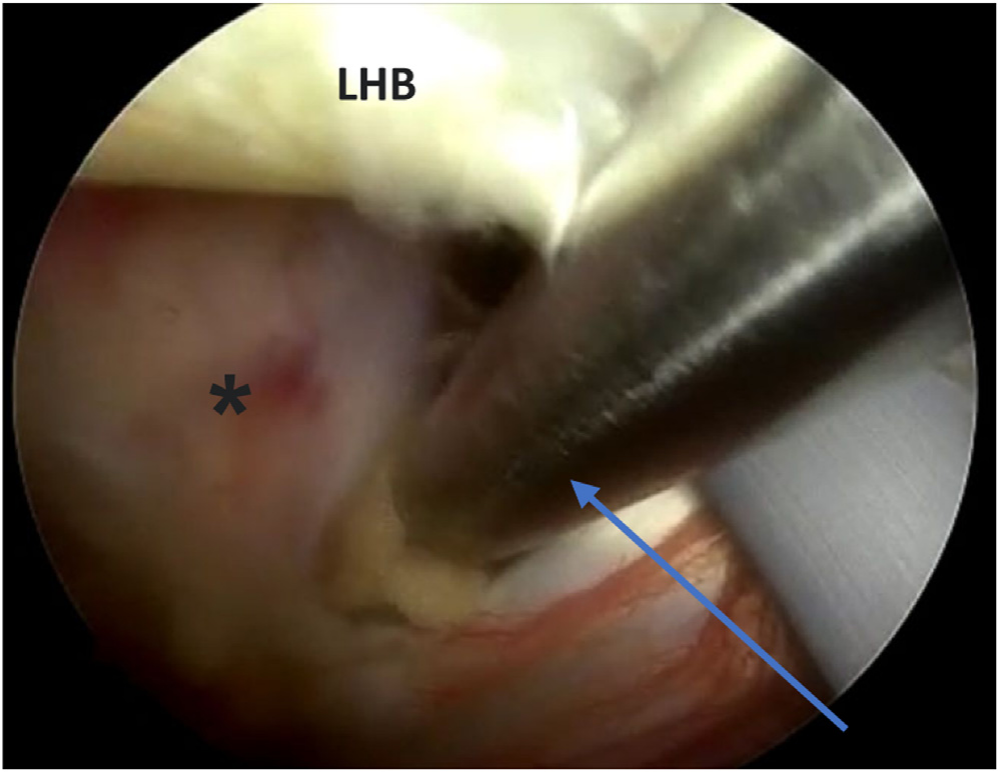
The patient is in a beach-chair position, and the right shoulder is shown. Viewing from the anterolateral portal, in the subacromial space. A periosteal elevator (arrow) is introduced from an accessory anteroinferolateral portal (biceps portal) to retract the LHB from its groove (asterisk) for anchor placement. (LHB, long head of biceps.)

After releasing the biceps tendon from its groove, a periosteal elevator is used to retract the biceps tendon away (Fig 6). The groove prepared with a rasp, and then the bicipital groove is drilled at its lowest part, just above the pectoralis major tendon, using a 3.9-mm drill bit. A shaver is then used to clear the drilled hole out of debris. Then, a PEEK (polyether ether ketone) Knotless Corkscrew Anchor, 3.9 mm × 11.2 mm (Arthrex), is inserted as distal in the groove. Thereafter, the biceps tendon is released from the periosteal elevator.

#### Suture Management

**Fig 7:**
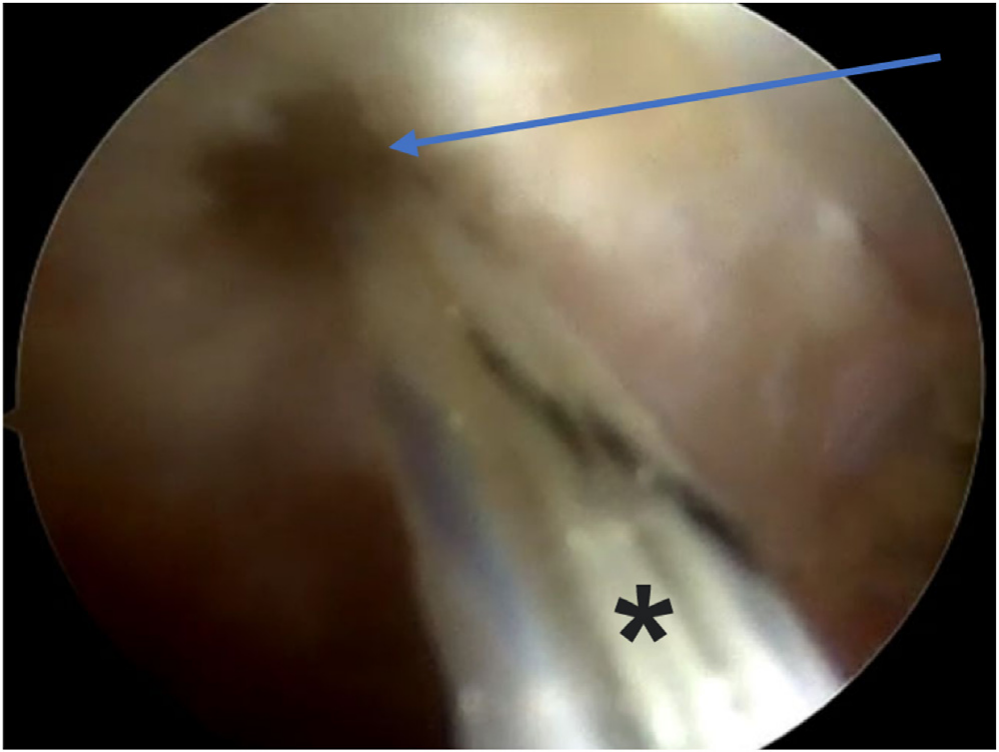
The patient is in a beach-chair position, and the right shoulder is shown. Viewing from the anterolateral portal, in the subacromial space. Drilling of the bicipital groove is done. An anchor (arrow) is placed from an accessory anteroinferolateral portal (biceps portal) with 3 FiberWires (asterisk) coming out from it.

**Fig 8:**
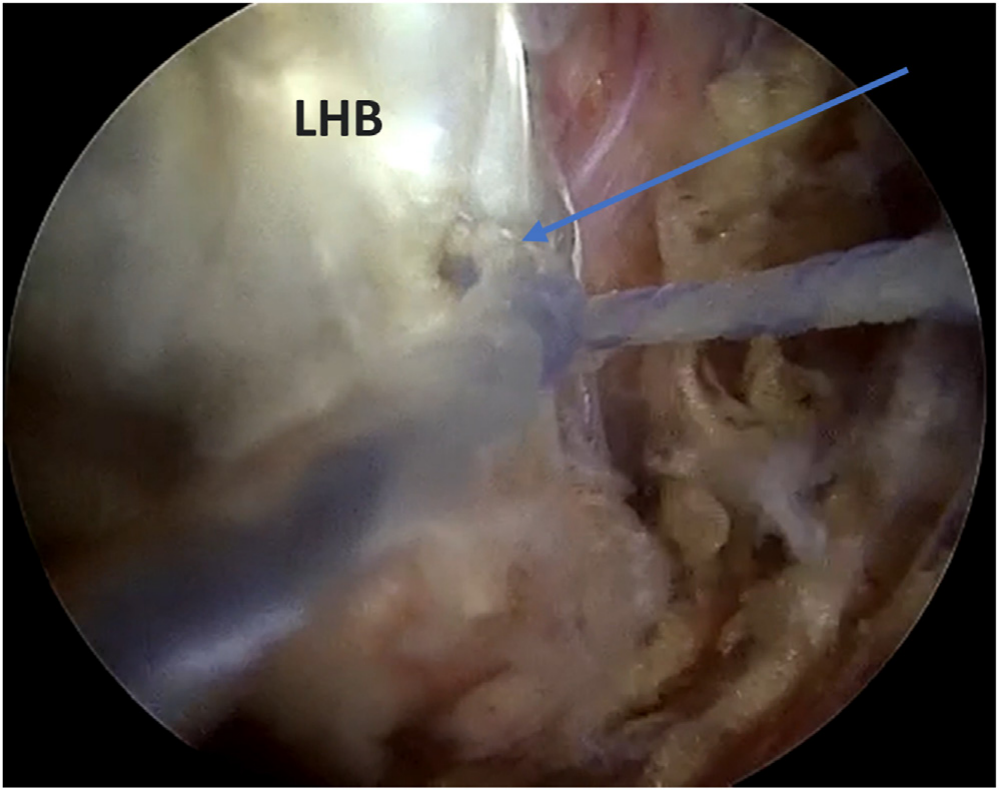
The patient is in a beach-chair position, and the right shoulder is shown. Viewing from the anterolateral portal, in the subacromial space. After the white FiberWire loop (shuttle loop) is shuttled into the substance of the LHB using a SutureLasso, the white FiberTape is pulled to shuttle the blue FiberWire (repair stitch) (arrow) into the substance of the biceps tendon. (LHB, long head of biceps.)

**Fig 9:**
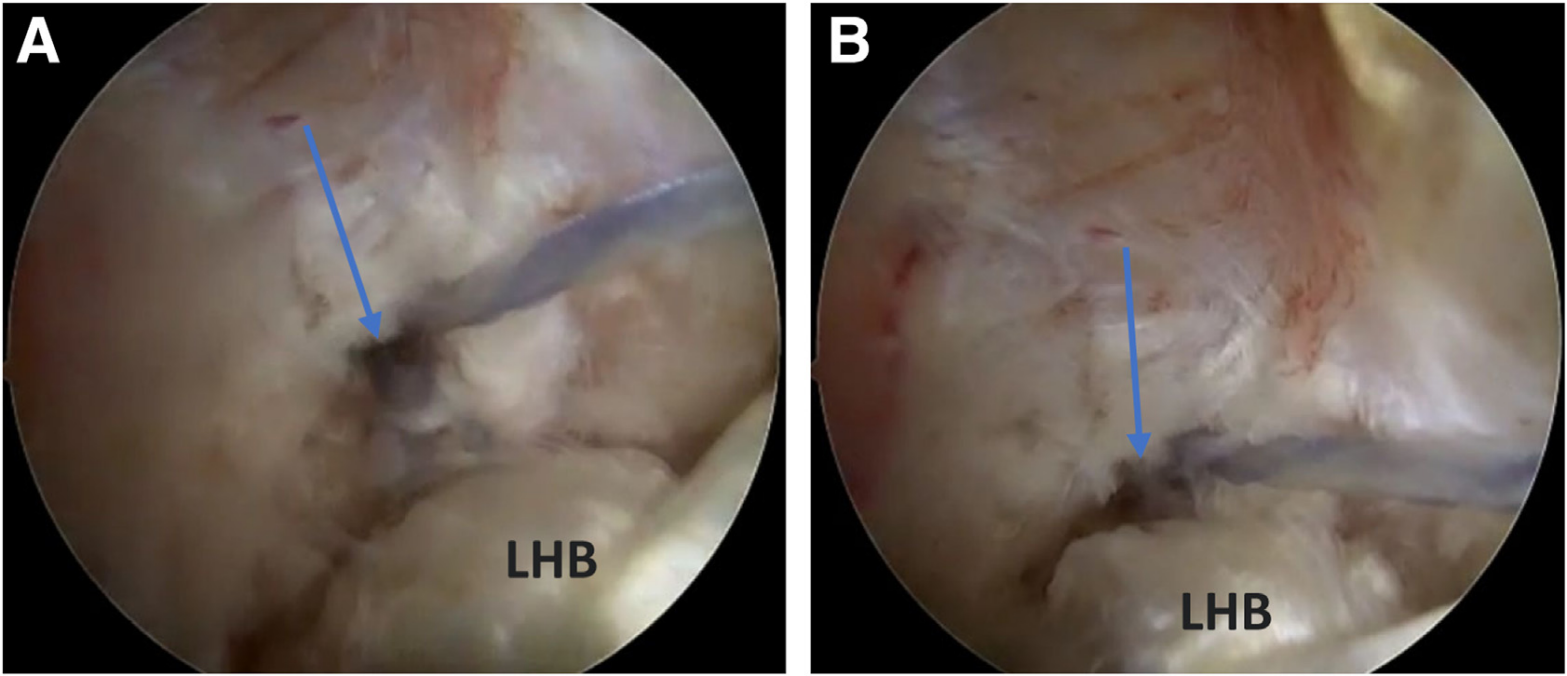
The patient is in a beach-chair position, and the right shoulder is shown. Viewing from the anterolateral portal, in the subacromial space. Starting with simple easy tensioning of the LHB with elbow into 20 to 25° of flexion. Then, (A) scope was passed posterior to the biceps tendon to view the anchor (arrow). (B) Final retensioning to the biceps tendon was done by pulling the blue wire (repair stitch) more. (LHB, long head of biceps.)

The anchor comes with 3 wires (Fig 7). A blue FiberWire (repair suture; Arthrex), a white FiberWire with a loop end (shuttle loop), and a white FiberTape (Arthrex). The blue wire (repair suture) is looped around the biceps tendon for one and a half turns. Then, a 45° SutureLasso (Arthrex) is used to go through the substance of the biceps tendon. A grasper is used to retrieve the lasso loop. The white FiberWire (shuttle loop) is shuttled through the lasso loop, and the lasso is removed. Now, the blue wire (repair suture) is passed through the white FiberWire loop (shuttle loop), making sure that the wires are moving freely and there's no soft-tissue bridge between them. Then, the 18-gauge spinal needle in the biceps tendon is removed, and the white FiberTape is pulled to pass the blue wire (repair stitch) in the biceps tendon substance (Fig 8). This will do indirect passage of the blue wire (repair stitch) through the biceps tendon. After that, we lock the repair suture into the anchor. At first, we start with simple easy tensioning of the LHB, then we place the elbow into 20 to 25° of flexion to make sure not to overtension the LHB. An arthroscopic retrieval is used to check the tension of the biceps tendon. Then, the scope is passed posterior to the biceps tendon, and final retensioning is done by pulling the blue wire (repair suture) again (Fig 9 A and B). A cutter is used to cut the blue wire. Also, with the help of a radiofrequency ablator or an arthroscopic scissor, the remaining prominent part of the biceps tendon is cut proximal to the anchor.

## Discussion

Controversies exist regarding the function of the LHB tendon on the glenohumeral joint, as some suggest it has multiple roles, such as being a depressor to the humeral head and contributing to glenohumeral joint stability.^7^^,^^8^ Pathologies in the LHB tendon involve a spectrum of diseases that range from tendinitis to degenerative tendinosis type of a process. Surgical treatment for LHB tendinopathies has been well described in the literature. However, the optimal surgical treatment is still debatable.

We believe arthroscopic extra-articular suprapectoral biceps tenodesis is an excellent option to address biceps pathologies, especially in active patients. With this technique, you also can address pathologies in the bicipital groove. This is due to the fact that this is a watershed area and prone to frictional attrition.

As overtensioning of biceps tenodesis can lead to tenodesis failure whereas undertensioning of the biceps can produce a pseudo-Popeye deformity.^10^, ^11^, ^9^ We describe an arthroscopic technique for distal suprapectoral biceps tenodesis using a knotless corkscrew anchor that allows the surgeon to do sequential and controlled tensioning on the LHB, which poses a greater challenge to the surgeon intraoperatively. Pearls and pitfalls of this technique are listed in Table 1.

**Table 1:** Pearls and Pitfalls

Step	Pearls	Pitfalls
1) Opening an accessory (biceps) portal.	A) Provides easy access to the bicipital groove to work in.	A) Placing the portal a bit anterior can cause difficulty in finding the groove. B) Needs an accessory portal.
2) Placing the biceps tendon in the lower part of the groove.	A) Better addresses the LHB pathology, especially in patients with groove disease.	A) Technically demanding to find the bicipital groove arthroscopic.
3) Looping the wire around the biceps tendon and using a lasso tool.	A) Passing the repair stitch around and through the biceps tendon gives a 2-point fixation to the biceps tendon. B) Doesn't require a cannula usage.	A) Needs special instruments to pass the suture, like a SutureLasso.
4) A finial retensioning of the biceps tendon.	A) Sequential tensioning is a good way to tension the biceps tendon to the anchor to provide more secure fixation. B) Knotless anchor usage.	A) Make sure to place the elbow into 20-25° of flexion to avoid overtensioning.

A few limitations of this technique include a possible learning curve for the surgeon to find the groove, and subpectoral pathologies are not addressed. The advantages and disadvantages of this technique are listed in Table 2.

**Table 2:** Advantages and Disadvantages

Advantages	Disadvantages
1) All-arthroscopic procedure	1) Onlay type of fixation
2) Suprapectoral and extra-articular tenodesis	2) Technically demanding
3) Use of knotless anchor	3) The need to use an accessory portal and extra instrumentation (SutureLasso)
4) Smaller drill hole	
5) Two-point fixation by looping the wire around and passing it through the tendon.	
6) Controlled retensioning of the LHB	
7) Address bicipital groove pathologies	

LHB, long head of biceps.

## Disclosures

The authors (Z.E.Q., M.M., A.J., B.E., W.A.) declare that they have no known competing financial interests or personal relationships that could have appeared to influence the work reported in this paper.

## Supplementary Data

Video 1 Arthroscopic suprapectoral retensioning biceps tenodesis using a knotless corkscrew anchor: this video shows a case example of a right shoulder of a patient in a beach-chair position viewed from the posterior portal. The following steps are included: (1) arthroscopic evaluation, (2) biceps tenotomy, (3) subacromial space preparation, (4) changing the viewing portal to an anterolateral portal, (5) opening the biceps portal, (6) localization of the long head biceps tendon in its groove, (7) insertion of knotless corkscrew anchor, (8) suture management, (9) suprapectoral biceps tenodesis, and (10) final retensioning of biceps tenodesis.
